# Oxali-palladium nanoparticle synthesis, characterization, protein binding, and apoptosis induction in colorectal cancer cells

**DOI:** 10.1007/s10856-023-06766-8

**Published:** 2024-01-11

**Authors:** Nasim Golestannezhad, Adeleh Divsalar, Farideh Badalkhani-Khamseh, Milad Rasouli, Arefeh Seyedarabi, Behafarid Ghalandari, Xianting Ding, Fatemeh Goli, Sander Bekeschus, Ali Akbar Moosavi Movahedi, Mahboube Eslami Moghadam

**Affiliations:** 1 Department of Cell & Molecular Sciences, Faculty of Biological Sciences, Kharazmi University, 49 Dr. Mofatteh Ave, 31979-37551 Tehran, Iran; 2 Department of Physical Chemistry, Faculty of Sciences, Tarbiat Modares University, Jalale-Al-Ahmad Ave, P.O. Box 14117-13116, Tehran, Iran; 3 Department of Physics, Kharazmi University, 49 Dr. Mofatteh Ave, Tehran, 15614 Iran; 4 Endocrinology and Metabolism Research Center, Tehran University of Medical Sciences, Jalale-Al-Ahmad Ave, 1411713137 Tehran, Iran; 5 Institute of Biochemistry and Biophysics (IBB), Tehran University, Tehran, 1417614418 Iran; 6 State Key Laboratory of Oncogenes and Related Genes, Institute for Personalized Medicine, School of Biomedical Engineering, Shanghai Jiao Tong University, Shanghai, 200030 China; 7 ZIK Plasmatis, Leibniz Institute for Plasma Science and Technology (INP), Felix-Hausdorff-Str. 2, 17489 Greifswald, Germany; 8 Chemistry & Chemical Engineering Research Center of Iran, Pajohesh Blvd,17th Km of Tehran-Karaj Highway, 1497716320 Tehran, Iran

## Abstract

This paper focuses on the synthesis of nano-oxali-palladium coated with turmeric extract (PdNPs) using a green chemistry technique based on the reduction in the Pd (II) complex by phytochemicals inherent in turmeric extract. PdNPs were examined and characterized using Field Emission Scanning Electron Microscopy (FESEM), Dynamic Light Scattering (DLS), Fourier Transform Infrared (FTIR), and Atomic Force Microscopy (AFM). Using different spectroscopic and molecular dynamics simulations, a protein-binding analysis of the produced nanoparticle was conducted by observing its interaction with human serum albumin (HSA). Lastly, the cytotoxic effects and apoptotic processes of PdNPs were studied against the HCT116 human colorectal cell line using the MTT assay and flow cytometry tests. According to the findings, PdNPs with spherical and homogenous morphology and a size smaller than 100 nm were generated. In addition, they can induce apoptosis in colorectal cancer cells in a dose-dependent manner with a lower Cc_50_ (78 µL) than cisplatin and free oxali-palladium against HCT116 cells. The thermodynamic characteristics of protein binding of nanoparticles with HSA demonstrated that PdNPs had a great capacity for quenching and interacting with HSA through hydrophobic forces. In addition, molecular dynamics simulations revealed that free oxali-palladium and PdNP attach to the same area of HSA via non-covalent interactions. It is conceivable to indicate that the synthesized PdNPs are a potential candidate for the construction of novel, nature-based anticancer treatments with fewer side effects and a high level of eco-friendliness.

**Graphical Abstract:**

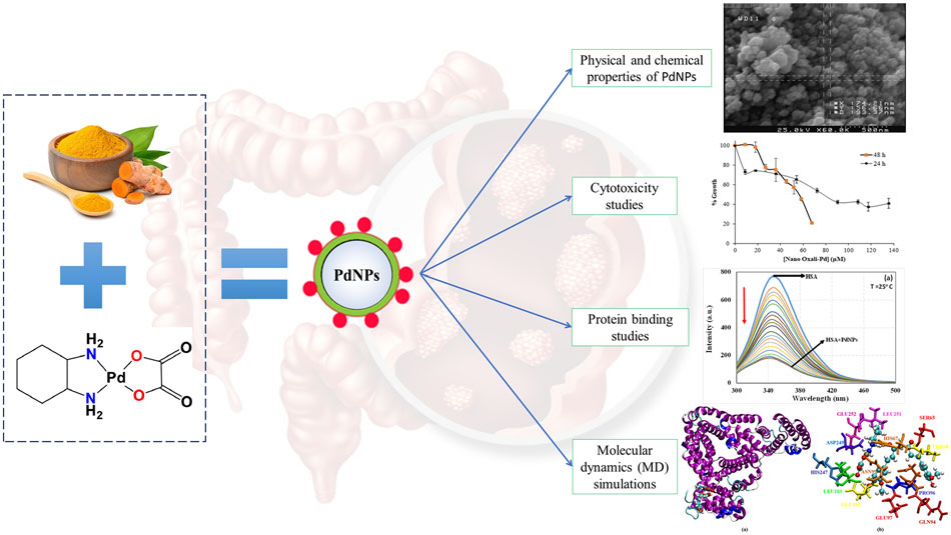

## Introduction

Cancer is a major threat to human health and a major issue for medical professionals and scientists. Annually, 1.09 million new cases and 700,000 deaths are attributed to colorectal cancer, making it the third most prevalent form of cancer worldwide and the world’s second deadliest type of cancer, creating a major economic burden around the world [1, 2]. Forecasts show that by 2030, there will be more than 2.2 million new cases each year, with an estimated 1.1 million deaths [2–4]. These staggering data demonstrate the urgent need to find an alternative treatment for this disease and novel methods to slow its spread [5].

Various multimodal approaches depending on tumor-related characteristics and patient-related factors are used for colorectal cancer treatments, including surgery, metastasis local ablation therapy, chemotherapy regimens (e.g., 5-fluorouracil, oxaliplatin, and irinotecan alone or concomitantly with targeted agents), radiation, immunotherapy, and targeted therapy [6, 7]. Conventional once-therapeutic agents are accompanied by severe side effects despite the fact that they have a positive effect in the short term [8, 9]. Therefore, it is vital to identify effective techniques to improve the survival and prognosis of colorectal cancer patients. Particularly, the primary concerns with successful cancer chemotherapy are its toxicity to normal tissues and the emergence of chemoresistance in colorectal cancer, which restricts its therapeutic application [4, 10].

Since roughly 5000 years ago, metal ions have been employed in medicine [11]. Metal-based compounds bind to many biological molecules, such as proteins, nucleic acids, hormones, and enzymes, making them indispensable for biological research [12]. Metal-based drugs have a notable impact on cellular growth and functions, such as gene expression and cell division [13]. The most important platinum-based chemotherapy agents include cisplatin, carboplatin, oxaliplatin, and nedaplatin. Palladium analogs have received the greatest interest recently since they frequently share structural similarities with platinum compounds [14]. Some palladium (II) complexes have been shown to be significantly more hazardous than platinum-based treatments against tumor cells, according to previous findings [15]. Although metal-based compounds are effective in treating malignancies, they come with several undesirable side effects and are therefore not suggested as first-line therapy [16].

Synthesizing metal-based nanomedicines using green chemistry is a promising and critically important field of research nowadays [17]. The biosynthesis of metal-based nanoparticles is currently being researched as a potential replacement for the use of physical and chemical substances in the production of nanoparticles [18]. It is crucial for pharmaceutical companies, particularly those working with nanomedicine, to use ingredients that are both natural and environmentally friendly [19, 20]. There have been several instances of palladium nanoparticles being synthesized using green chemistry methods such as *Cinnamon zeylanicum bark* [21]*, C. Camphora leaf* [22]*, Curcuma longa tuber* [23]*, banana peel* [24]*, Rosa canina fruit* [25], and *Stachys lavandulifolia* [26].

On the other hand, traditional chemical and physical approaches to nanoparticle synthesis are hazardous and harmful to ecosystems. The biocompatible synthesis of nanoparticles with distinct sizes and shapes was difficult in biomaterial research until recently, when nanoparticle biosynthesis was revealed to be successful [27]. The primary goal of creating green nanoparticles is to reduce the quantity of toxic chemicals generated while also preventing environmental contamination. Reducing metal groups into targeted nanoparticles can be accomplished through natural products and plant extracts with antioxidant or reducing characteristics. Plant, bacterial, and fungal nanomaterials have numerous practical uses in engineering, medicine, and industry [28]. When making nanoparticles, it is important to use non-toxic ingredients to keep them stable and a safe solvent medium for the environment [27, 28].

The rhizome of the plant Curcuma longa is used to make turmeric, a golden herb that has been used for centuries as a source of flavor and color. Some illnesses, including infection, hepatitis, liver issues, and even cancer, were treated with this natural remedy in the past. It is processed to remove several chemical components, including polyphenols, terpenes, diterpenes, sterols, and alkaloids. Curcumin, or turmeric, has been shown in cell-based research to inhibit infection, cancer, and mutation. Curcumin is the most interesting component of turmeric. Hence, it makes up 2–5% of the extract [29].

Previous reports have shown that upon administration of nanoparticles into the systemic circulation, they can interact with plasma proteins and form protein corona [30]. Corona formation and composition have important effects on the toxicity and internalization of synthesized nanoparticles. Additionally, the interaction between nanoparticles and plasma proteins alters the surface functionality of nanoparticles and can affect their translocation behavior in biological systems [30, 31]. Therefore, there is an urgent need to understand the molecular mechanisms of nanoparticle interactions with plasma proteins (nature of interactions, interaction forces, binding sites, and affinity and their implications) and other biological functions to improve the design and handling of nanomaterials in a physiological environment [32, 33].

Human serum albumin (HSA) is one of the most abundant and major transport and model proteins in blood plasma for protein-binding research [32]. Studying the interactions of nanoparticles with HSA is critical to designing specific, more efficient, and less toxic drugs [30–32]. Thus, this research utilized a green synthetic technique to synthesize PdNPs from turmeric extracts. The PdNPs’ biological activities were next studied by determining whether or not they were carcinogenic to HCT116 cell lines and whether or not they interacted with human serum albumin (HSA). We also performed all-atom molecular dynamics (MD) simulations to assess the dynamical behavior of HSA:oxali-Pd and HSA:PdNP complexes, where PdNP is an oxali-Pd molecule coupled to a curcumin molecule. We believe that our research would help comprehend the safety of green synthesized nanoparticles when they are injected into the blood for different biomedical goals, such as drug delivery.

## Material and methods

### Materials

Both the HSA (>99% purity) and the trypsin were purchased from Sigma Co., US. Merk Co., Germany, provided chemicals such as MTT powder (3-(4,5-dimethylthiazol-2-yl)-2,5-diphenyl tetrazolium bromide), EDTA, Trypan Blue, PBS, Sodium Pyruvate, and NaHCO_3_. Gibco, UK supplied the 100 IU/mL penicillin, FBS, 100 µg/mL streptomycin, and DMEM/L-Glutamine. The oxali-palladium was produced in the lab using the established procedure [34]. A 5 mM NaCl solution was used to dissolve the oxali-palladium, and the pH was brought up to 7.0. Double-distilled water or de-ionized water was used to prepare the solutions.

### Plant extract preparation and PdNPs synthesis using turmeric extracts

To obtain turmeric extract, 1 g of turmeric powder was dissolved in 25 mL of ethanol and incubated for 6 h at 30 °C in a shaking incubator. The extract was then placed into a Petri dish and allowed to dry until the impurities were separated. For the synthesis of nanoparticles, 0.015 g of the turmeric extract and 3 mL of oxali-palladium solution (2 mM) were dissolved in 15 mL of ethanol. The solution was placed in a shaking incubator at 50 °C and 200 rpm for 24 h. Changes in color solution showed the synthesis of PdNPs. In this case, the solution color change from yellow to orange revealed the completion of the nanoparticle synthesis process. Nanoparticles are created in a centrifuge at 6000 rpm for 30 min. The supernatant was collected, and 3 mL of double-distilled water was added to the precipitate. The lyophilized solution was stored for further use in characterization, protein binding, and cytotoxicity analyses.

### Nanoparticles characterization

Dynamic Light Scattering (DLS) (Brookhaven Instruments Corporation, USA) was utilized to measure the size and zeta potential of NPs. First, the solution containing the green-synthesized NPs was sonicated for 10 min to facilitate homogenization and separation of the nanoparticles. The lyophilized powder was then reconstituted with double-distilled water. Zeta potential analysis was used to ascertain the nanoparticles’ stability. Atomic Force Microscopy (AFM; Veeco Instruments) and Field Emission Scanning Electron Microscopy (FE-SEM) were utilized to ascertain NP morphology and size, respectively. The NPs’ encapsulating chemical groups were investigated using infrared (IR) absorption spectroscopy. PdNPs, turmeric extract, and oxali-palladium solutions were used as examples [29].

### Inductively coupled plasma-atomic emission (ICP)

Through inductively coupled plasma-atomic emission spectroscopy, the spectra of elements are studied and recorded, revealing the elements’ real composition. This technique allows for identifying the total number of components and their concentration [30]. The synthesis solution, which contains PdNPs, was centrifuged, and the supernatant was tested by the ICP method to quantify the amount of free oxali-palladium, which does not participate in the nanoparticle synthesis reaction. The amount of encapsulated oxali-palladium was determined by calculating the concentration difference between primary and free oxali-palladium. The purpose of determining the encapsulated palladium concentration is to investigate the actual concentration of nanoparticles accurately in cellular studies.

### Cell culture

The human colorectal tumor cell line HCT116 was procured from the Pasteur Institute of Iran for cytotoxicity investigation. In a 5% CO_2_ incubator at 37 °C, the cell line was cultured in DMEM media supplemented with 10% fetal bovine serum (FBS), 100 IU/mL penicillin, and 100 µg/mL streptomycin.

### Viability assay using MTT

In a 96-well plate, cells were ‘plated at a density of 15,000 cells per well and then cultured for 24 and 48 h at 37 °C. Twenty-four hours were spent at 37 °C in a CO_2_ incubator with the plate. The cells were treated with turmeric extract, free oxali-palladium, and nano-oxali-palladium at various doses. In healthy cells, the mitochondrial enzyme dehydrogenase converts the yellow color of MTT to violet formazan crystals, which is the basis for Mosmann’s MTT assay [35]. Turmeric extract (0–120 mg/mL), PdNPs (0.0–145 µM), and free oxali-palladium (0.0–1500 µM) were added to the cells, and incubation continued for 24 and 48 h. In this experiment, oxaliplatin served as a “positive” control. After a period of 24 h, the supernatant was removed from the well. Then, 100 µL of RPMI medium and 5 µL of MTT (5 mg/mL) dye were poured into each well. Following this, the dish was tightly wrapped in foil and placed in the incubator for 2 h. After adding 50 µL of DMSO to each well, they were shaken for 10 min at 37 °C. The Cc_50_ parameter corresponds to the concentration of the drug that induces 50% mortality in cancer cells, and it was computed by measuring the absorption of the MTT dye at 570 nm and plugging the results into the following Equation (Eq. (1)).

1 $$Cell\,survival( \% )=\frac{{A}_{treated}}{{A}_{control}}\times 100$$

where *A*_treated_ is the absorbance of the treated cells, and *A*_control_ is the absorbance of the control cells.

### Apoptosis analysis

Apoptosis of HCT116 colon cancer cells was measured by flow cytometry using an Annexin VFITC/PI staining kit, per the manufacturer’s instructions, to determine how PdNPs cause cell death [36]. In this study, six-well plates were seeded with 8 × 10^5^ HCT116 cells per well. The cells were treated with 78 µM of nano-oxali-palladium after 24 h of culture, after which the supernatant media was discarded. Cell suspensions were centrifuged after treatment. Subsequently, the plates were washed in 1 µM of cold phosphate-buffered saline (PBS). The cells were then treated with 100 µL of binding buffer, 5 µL of Annexin V-FITC solution, and 5 µL of propidium iodide (PI) for 20 minutes in the dark at room temperature. After adding 400 µL of 1× binding buffer to the samples, the results were analyzed. A FACSCalibur and the program Cell Quest (Becton Dickinson, USA) were used to complete the data analysis.

### Statistical analysis

The findings of the cytotoxicity testing were analyzed using SPSS-19 software. A one-way analysis of variance was utilized to identify statistical differences. The mean ± standard error of the mean was used to characterize the findings (Post Hoc, LSD Test). Each experiment was carried out in triplicate. At *P* < 0.05, differences were deemed significant.

### Protein binding studies

Fluorescence spectroscopy (Varian Co., Australia) was used to investigate the biophysical mechanism of PdNPs-HSA interactions at 25 and 37 °C in the absence and presence of varying amounts of PdNPs. When the protein was excited at 295 nm, fluorescence emission spectra were taken between 300 and 500 nm. Changes in HSA’s fluorescence intensity were also measured.

### Molecular dynamics (MD) simulations

By running all-atom MD simulations in GROMACS 5.0, we investigated the dynamic behavior of the HSA: oxali-Pd and HSA:PdNP complexes [10.1016/j.softx.2015.06.001]. VMD 2.9 program was also used to visualize the MD trajectory and to analyze the simulated system [10.1016/0263-7855(96)00018-5]. The atomic structure of human serum albumin, determined by X-ray crystallography, can be accessed in the Protein Data Bank (PDB) database at the RCSB. The corresponding PDB ID is 4L8U [10.1016/j.bbagen.2013.06.032]. For atomistic MD simulation of HSA, the AMBER 99SB force field was implemented to determine bond lengths, bond angles, proper and improper dihedrals, and van der Waals and electrostatic forces [10.1016/S0065-3233(03)66002-X]. Using GaussView 5.0, the first models of oxali-Pd and curcumin (CUR) molecules were constructed. For geometric optimizations, the B3LYP (Becke, 3-parameter, Lee-Yang-Parr) functional was utilized. When simulating the physicochemical properties of small molecules, the B3LYP functional is a proven method with excellent performance in practically all areas of chemistry. In order to describe the chemical bonds between the carbon, nitrogen, oxygen, and hydrogen atoms, the 6–31 G(d) valence double-zeta polarized basis set has been used, while the lanthanum atom in the Pd atom was subjected to LANL2DZ ECP. DFT computations were performed entirely in Gaussian 03 [Frisch, M. J. “Gaussian 03 Rev. 01.” http://www.gaussian.com/ (2004)]. We have used the MCPB program, a python-based metal center parameter builder available in AmberTools that is compatible with a wide range of AMBER force fields, to obtain the necessary parameters for the simulation of oxali-Pd. To make the MCPB data suitable for use in GROMACS, the ACPYPE tool was utilized to convert the output data [10.1186/1756-0500-5-367]. The partial charges of proteins and ligands were determined using a restrained electrostatic potential (RESP) method. The HSA:oxali-Pd and HSA:PdNP complexes were constructed from the starting structures obtained from the molecular docking technique. To foretell how oxali-Pd and PdNP (containing one oxali-Pd and one CUR molecule) attach to the protein, we performed molecular docking calculations with the help of AutoDock 4.2 software [10.1002/jcc.21256]. All HSA:oxali-Pd and HSA:PdNP complexes had 75, 40, and 75 grid points in the x, y, and z directions, respectively. The box was positioned at the protein’s mass center, and the grid spacing was adjusted at 1 Å. Finally, the conformations with the energy score were selected for MD simulations. The particle mesh Ewald approach and a 12 Å radius cutoff were applied to account for long-range electrostatic and short-range non-bonded forces, respectively. Cubic solvation boxes filled with the TIP3P water model and a 10 Å solvation shell were used to position the HSA:oxali-Pd and HSA:PdNP complexes, respectively. To neutralize the system, the correct number of chloride ions (Cl^−^) was supplied to the simulation box. To simulate the electrolytes of the human body, 0.145 M of salt (Na^+^Cl^−^) was introduced to the system. In all directions, periodic boundary conditions were applied. The steepest descent technique was employed to conduct energy minimization, followed by the conjugated gradient approach. Using the V-rescale coupling approach, the simulation system was then cooled to 310 K under a 1 ns canonical (NVT) ensemble. During 1 ns of NPT simulations using the Parrinello–Rahman coupling method, the pressure was maintained isotropically at 1.0 bar. After completing two equilibration stages, the position restrictions were released, and 100 ns of production MD runs were conducted.

## Results and discussion

### Preparation of nano-oxali-palladium using turmeric extracts

When oxali-palladium was added to the turmeric ethanolic solution, a color change indicated that nano-oxali-palladium had formed and that metallic Pd had been reduced to Pd nanoparticles. After adding oxali-palladium and incubation for 24 h, a yellow turmeric solution in ethanolic solvent turned orange. Furthermore, a comparison of the 200–600 nm absorption spectra of free oxali-palladium and nano-oxali-palladium revealed that the highest absorption of free oxali-palladium occurred at a wavelength of 220 nm. However, nano-oxali-palladium failed to exhibit maximal absorption. Previous studies concur that the reduction in metal palladium ions and their subsequent conversion to palladium nanoparticles is represented by a shift in the highest absorption peak in nano-oxali-palladium [37, 38]. Arsiya et al. reported that a color change from yellow to dark brown was observed after the reduction reaction (conversion of Pd (II) ions in the PdCl_2_ sample to PdNPs at a peak range of 370–440 nm), which is in good agreement with our results [39]. This phenomenon is due to surface plasmon resonance (SPR) because metallic nanoparticles have free electrons on their surface [40].

### Characterization studies of PdNPs

**Fig. 1:**
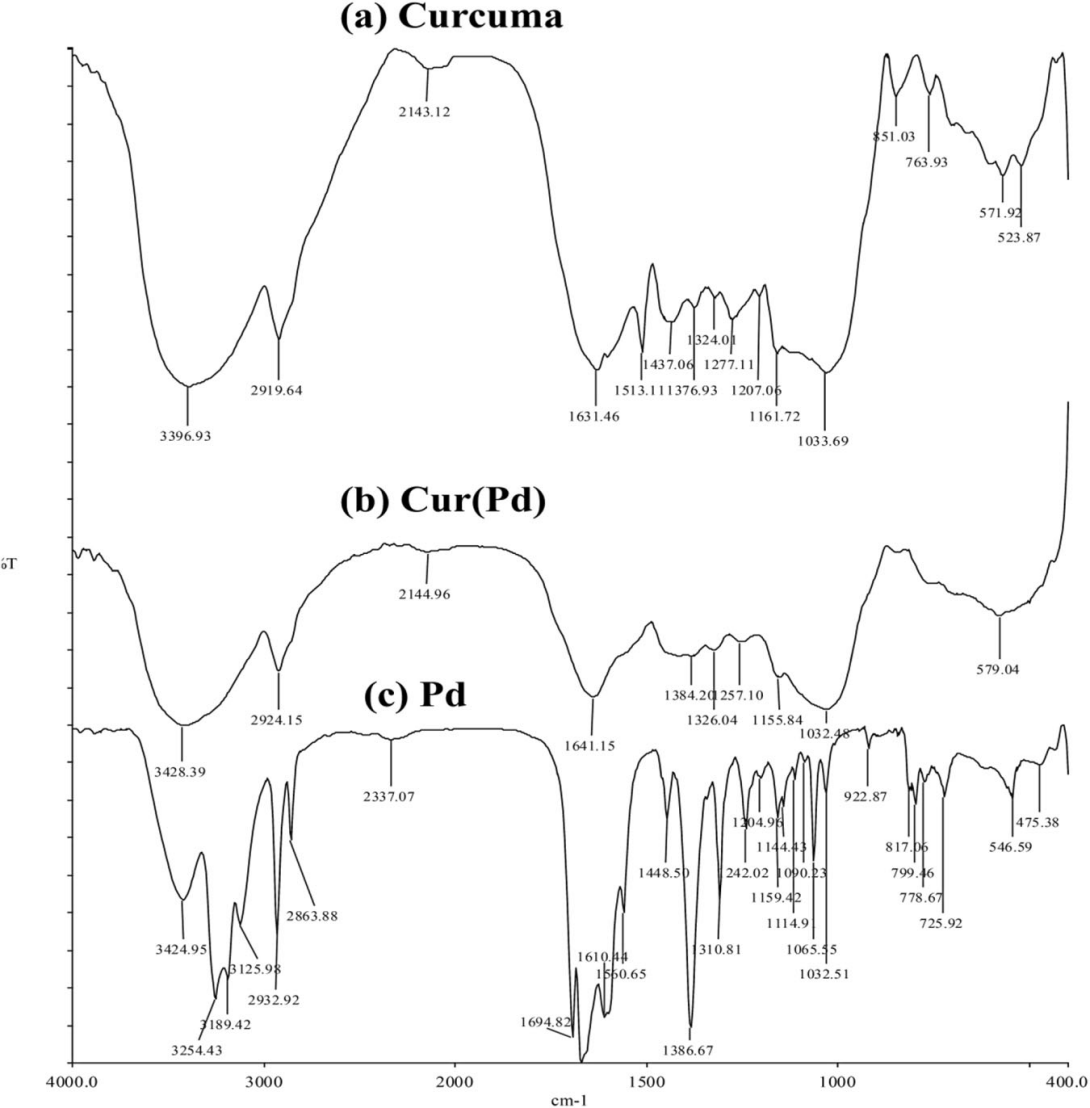
FTIR diagrams of (**a**) turmeric extract, (**b**) nano-oxali-palladium coated with turmeric extract, and (**c**) free oxali-palladium. Free oxali-palladium (**c**) and nano-oxali-palladium (**b**) display alkyne functional groups at 2144.96 cm^−1^ and 1641.15 cm^−1^, respectively. Common functional groups in nano-oxali-palladium (**b**) and turmeric extract (**a**) show that nano-oxali-palladium is coated with turmeric extract

**Fig. 2:**
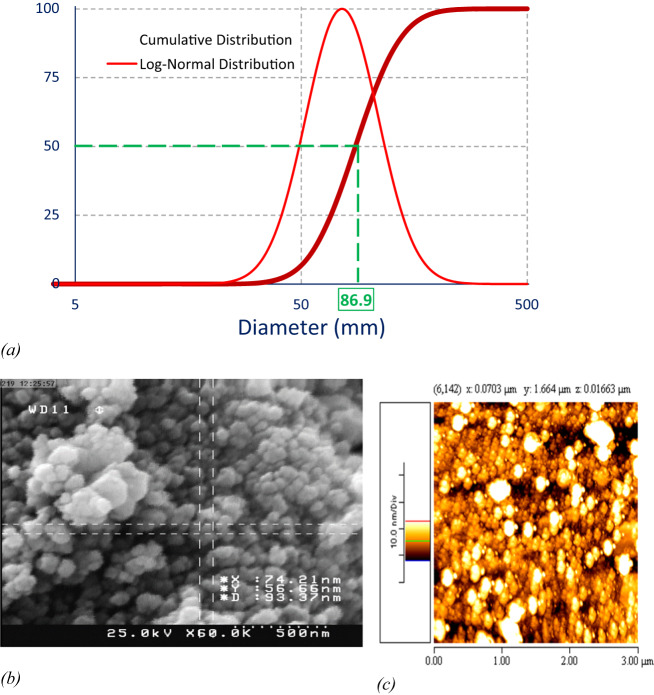
DLS diagram of (**a**) PdNPs; (**b**) Field Emission Scanning Electron Microscopy of the green synthesized nano-oxali-palladium; (**c**) Atomic Force Microscopy of nano-oxali-palladium

FTIR analysis has been utilized to detect the newly synthesized functional groups in nanoparticles [41]. FTIR spectra of turmeric extract are displayed (Fig. 1), free oxali-palladium, and nano-oxali-palladium coated with turmeric extract. In contrast to free oxali-palladium, nano-oxali-palladium displays alkene and alkyne functional groups at 2144.96 cm^−1^ and 1641.15 cm^−1^, respectively. Coating the nano-oxali-palladium with turmeric extract is evidenced by the functional groups shared by the two substances. The distinctions between the most frequent functional groups and the encapsulated oxali-palladium functional groups are shown (Table S1). The FTIR spectrum of the *Z. officinale and C. longa* rhizome extracts has shown a maximum absorption peak at 1387 cm^−1^ in the green synthesis of AgNPs [42, 43]. Other studies show that the phytochemicals and proteins observed in the FTIR spectrum reveal that the amide and amine groups present in F. *decipiens* (the plant extract that is applied to synthesized PdNPs) may be involved in the bio-reduction reaction of PdNP synthesis [44]. The cumulative distribution and log-normal distribution diagrams of DLS analysis (Fig. 2) show that the mean diameter of PdNPs is 86.9 nm, indicating that 50% of synthesized particles are in the 10–100 nm range. Additionally, the Zeta potential, which shows the surface load of the particles, was analyzed by DLS. The Zeta potential for PdNPs was obtained at −22.77 mV. More negative or positive zeta potential means that the particles have less affinity to be agglutinated. Electron microscopy studies were performed to confirm the DLS results further and investigate the homogeneity of synthesized Pd nanoparticles. Zeta potential values of nanoparticles between >25 mV and <−25 mV show stable structure [45]. The results of FESEM (Fig. 2b) and AFM (Fig. 2c) show that PdNPs are not only homogeneous and well dispersed spherically shaped but also have a regular shape with an average diameter of around 90 nm, which agrees well with DLS data. Different kinds of palladium nanoparticles have been investigated in Kanchana et al. studies, which showed that PdNPs are polydisperse and their size is between 60 and 100 nm [46].

### Encapsulation efficiency of oxali-palladium

Using the ICP method, the concentration of free oxali-palladium was determined to be 0.1 mM. Prior to nanoparticle creation, the main concentration of oxali-palladium in solution was 0.33 mM; therefore, the concentration difference between the primary or total and free oxali-palladium concentration is referred to as encapsulated oxali-palladium. The measured encapsulation value is 0.23 mM. This result indicates that the encapsulation efficiency (EE) was 54%. According to these findings, more than half of the oxali-palladium was encapsulated in PdNP.

### Cytotoxicity studies

**Fig. 3:**
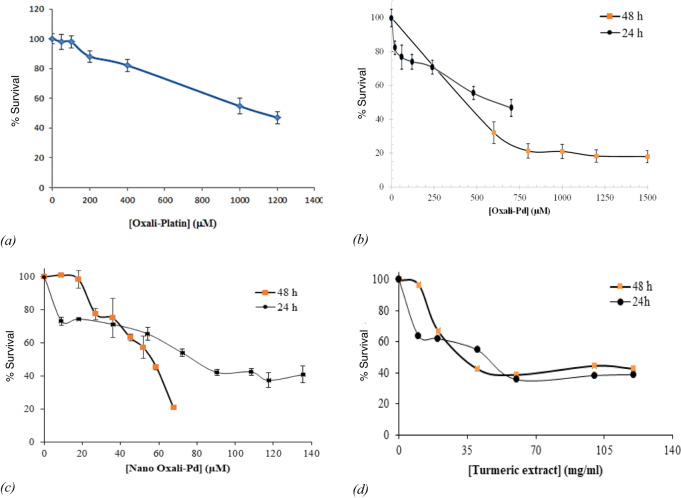
MTT test results of HCT116 cells after treatment with (**a**) oxaliplatin (0–1200 µM), (**b**) oxali-palladium (0–1500 µM), (**c**) PdNPs (0–145 µM), and (**d**) crude turmeric extract (0–120 mg/mL) after 24 and 48-h incubation times at 37 °C in a CO_2_ incubator

**Fig. 4:**
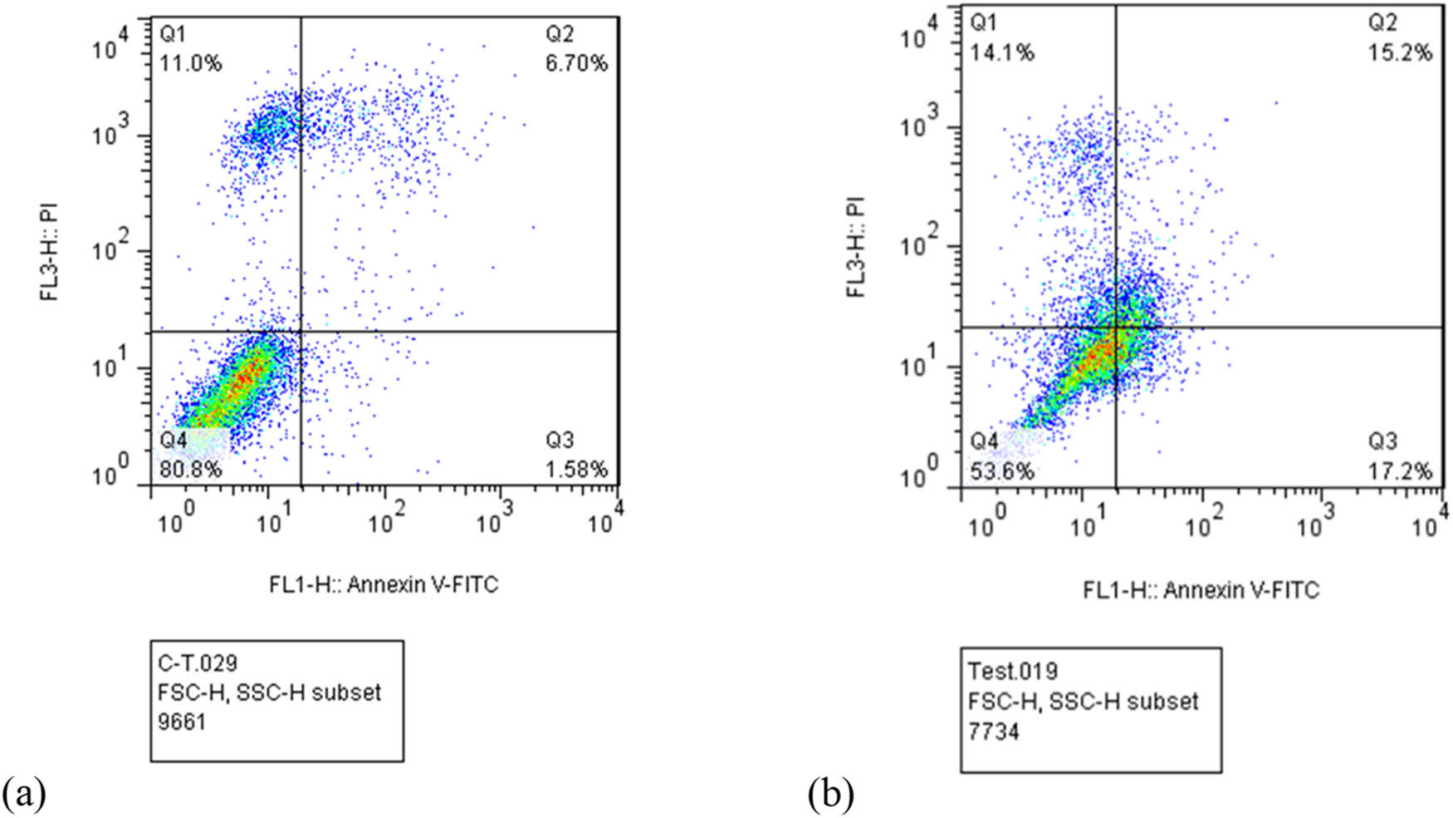
Common quadrant analysis of Annexin V-FITC/propidium iodide flow cytometry of HCT116 cells for (**a**) control sample and (**b**) treated with PdNPs at a concentration of 78 µM

The MTT assay was employed for the quantitative analysis of cytotoxicity. After 24 and 48 h of incubation, the MTT test was performed to see whether or not synthesized PdNP had any effects on the human colorectal cancer cell line HCT116. PdNPs can cause dose- and time-dependent cell death in HCT116 cells (Fig. 3b–d). The percent of cell survival of cancer cells is greatly stunted by increasing the amount of oxali-palladium, PdNP, and turmeric extract. HCT116 also exhibits a concentration- and time-dependent inhibiting activity in its growth inhibition curve. Cc_50_ results from MTT assay plots revealed that PdNP induced significantly higher mortality in HCT116 cells compared to exposure to free oxali-palladium at lower dosages (Fig. 3b–d and Table 1). Additionally, PdNPs are significantly more hazardous to the HCT116 cell line than the commonly used chemotherapeutic agent oxaliplatin (1100 µM after 24 h of incubation) **(**Fig. 3a–d). These PdNPs were shown to be 14x times more toxic than oxaliplatin and 7.7× times more lethal than free oxali-palladium when tested against the HCT116 cancer cell line. Furthermore, the Cc_50_ value of crude turmeric extract was determined to be 45 and 32 mg/mL after 24 and 48 h of incubation, respectively, against cancer cell lines (Fig. 3d). In the current investigation, 1 mg/mL of crude turmeric extract was used to synthesize PdNP, which is significantly lower than the cytotoxic concentration of the extract. Another study using MTT assays showed that the synthesized oxali-palladium nanoparticles using *Agaricus bisporus* produced a dose- and time-response inhibition on the growth of HCT116 cell lines [47–49]. Moreover, in the other study, the MTT assay demonstrated that PdNPs have an excellent cytotoxic effect against cancer cells. A maximum growth exhibition of 79% was observed for the maximum dose [50]. To examine the mechanism by which PdNP induces cell death in the HCT116 human colorectal cancer cell line, flow cytometry analysis was performed in the absence and presence of PdNP (at Cc_50_ concentration for 24 h). The majority of cells in the control group of PdNPs-untreated HCT116 cells were alive, as shown by flow cytometry (Fig. 4a), except for 6.70% late apoptotic and 11% necrotic cells. 15.2% late apoptotic, 17.2% early apoptotic, 14.1% necrotic, and 53.6% viable cells were detected by flow cytometry at a concentration of 78 µM, which is the Cc_50_ value of PdNPs after 24 h of incubation (Fig. 4b). In this investigation, total early and late apoptosis was found to be 32.4% in samples treated with PdNPs, which was significantly greater than the control sample (8.28%). At a concentration of 78 µM, PdNPs can trigger apoptosis in HCT116 human colorectal cancer cells.

**Table 1:** Cc_50_ values for oxpd, oxpdNps, oxpt, and turmeric extract after 24 and 48 h of incubation

Compounds	Cc_50_ After 24 h	Cc_50_ After 48 h
Oxpd	600 (μM)	433 (μM)
Oxpd NPs	78 (μM)	57 (μM)
Oxpt	1100 (μM)	–
Turmeric extract	45 (mg/mL)	32 (mg/mL)

### Protein binding studies

**Fig. 5:**
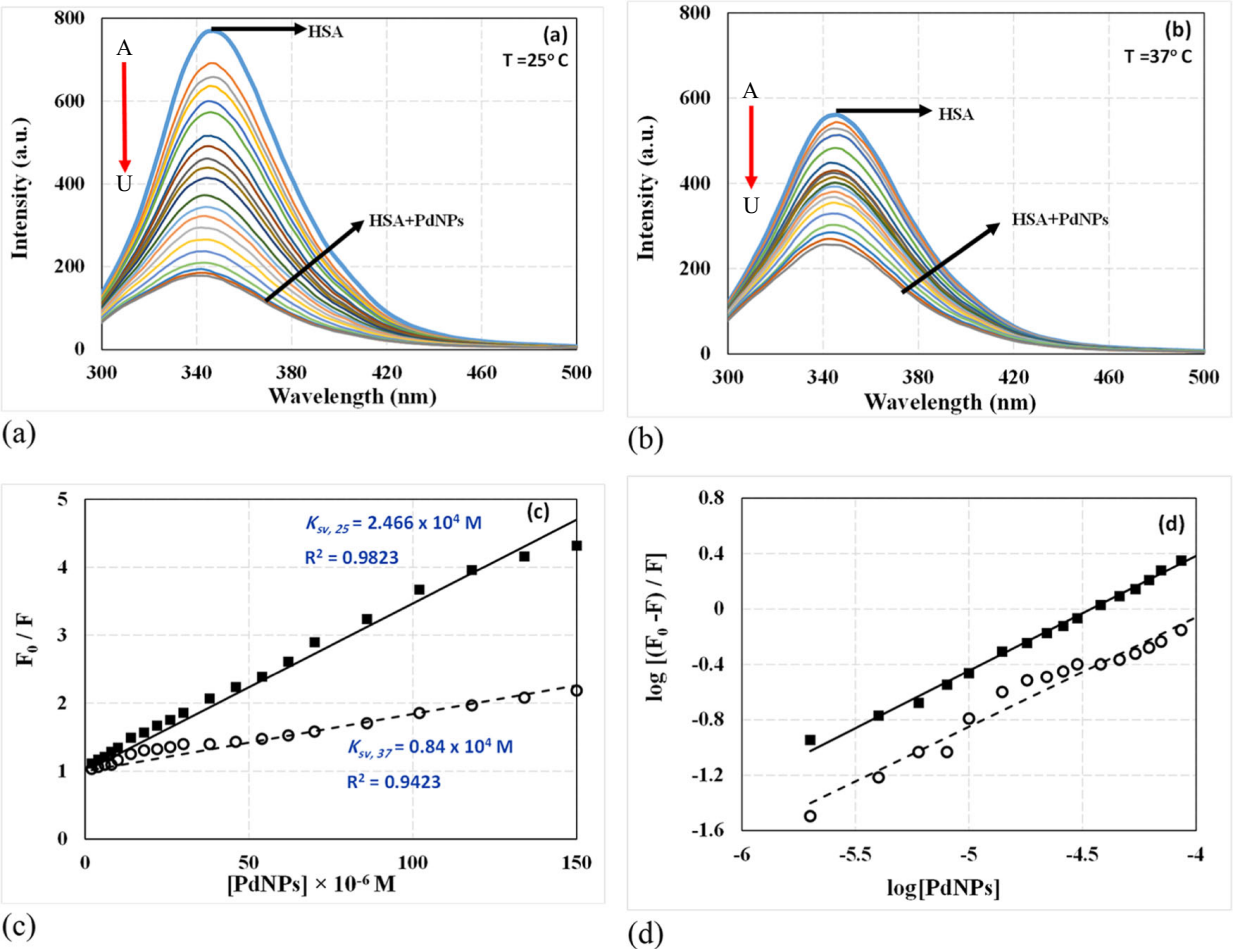
Fluorescence spectra of HSA (0.1 µM) upon addition of various concentrations of PdNPs (0 (A), 2 (B), 4 (C), 6 (D), 8 (E), 10 (F), 14 (G), 18 (H), 22 (I), 26 (J), 30 (K), 38 (L), 46 (M), 54 (N), 62 (O), 70 (P), 86 (Q), 102 (R), 118 (S), 134 (T), 150 (U) µM) from up to down in Tris-HCL buffer (pH 7.5) at (**a**) 25 °C and (**b**) 37 °C. The arrow indicates the sequence of spectral changes upon increasing PdNP concentration, corresponding to a decrease in intensity. **c** Linear Stern–Volmer plot of *F*_0_/*F* vs. [Q] at 25 °C (black square) and 37 °C (white circle). **d** Scatchard plot of log [(*F*_0_−*F*)/*F*] vs. log [Q] at 25 °C (black square) and 37 °C (white circle), pH 7

Fluorescence spectroscopy is an effective method for investigating the conformation, dynamics, structure, and binding properties of macromolecules (enzymes, proteins, nucleic acids) and ligands (e.g., metals, drugs, and other substrates) in solutions [51]. It has been reported that Trp^214^ in subdomain IIA of the HSA is an excellent marker for fluorescence studies since it is the most intense of all the intrinsic aromatic fluorophores in the HSA molecule [51]. Hence, we used fluorescence spectroscopy to examine HSA and PdNP’s interaction at both ambient and physiologic temperatures in the present work. The presence and absence of PdNP at 25 and 37 °C are shown (Fig. 5a, b), demonstrating how the intrinsic emissions spectra of HSA are between 300 and 500 nm at different concentrations. The protein’s fluorescence emission is diminished and muted as the concentration of nanoparticles in the protein solution increases over time (Fig. 5c, d). Two distinct types of quenching mechanisms, dynamic and static, are distinguished by the ranges of temperature and viscosity at which they operate. The diffusion coefficient increases as the temperature rises, which is important for dynamic quenching. Consequently, the temperature rises as the quenching constant grows. When the temperature is lowered, however, the static quenching constantly drops. The Stern–Volmer equation (Eq. (2)) was used to investigate the quenching mechanism [51]:

2 $$\frac{{F}_{0}}{F}=1+{K}_{SV}[Q]$$

where *F*_0_ and *F* denote the intensity of HSA’s fluorescence emission in the absence and presence of PdNP, respectively. KSV is the Stern–Volmer quenching dynamic constant, and [*Q*] is the total quencher concentration (PdNP in this case) [52, 53]. Stern–Volmer plots of *F*_0_/*F* versus quencher concentrations [*Q*] at 25 and 37 °C are shown (Fig. 5c). At temperatures of 25 and 37 °C, the Stern–Volmer constants resulting from the interaction of HSA-PdNP were calculated to be 23.0 × 10^−3^ and 8.4 × 10^−3^ M^−1^, respectively. These data demonstrated that increasing the temperature from 25 to 37 °C reduced the values of the Stern–Volmer constants. Hence, it can be assumed that PdNP turns off through complex formation between a ligand and a protein, not through dynamic collisions. Equation (3) was utilized to determine the binding parameters of PdNP’s interaction with the carrier protein HSA, including the equilibrium binding constant (*K*_b_) and the number of binding sites (*n*) (Eq. (3)) [54].

3 $$Log\left(\frac{{F}_{0}-F}{F}\right)=Log({K}_{b})+nLog[Q]$$

The log [(*F*_0 _− *F*)/*F*] versus log [*Q*] plots at 25 and 37 °C are depicted (Fig. 5d). The plot was analyzed using the linear least squares method, which showed the number of binding sites (*n*), the slope of the nanoparticle regression curve, and the binding constants (*K*), which were found by taking the log of the intercept (the log *K*). One PdNP molecule and one HSA molecule interacted at both temperatures, with the *n*-value for PdNP on HSA being close to 1 at both temperatures (Fig. 5b). Additionally, estimated binding constant values at two temperatures demonstrate that the affinity of PdNP for protein diminishes as the temperature rises from 25 to 37 °C (binding constant values were 0.50 × 10^4 ^M^−1^ and 0.09 × 10^4 ^M^−1^, respectively). Previous investigations using fluorescence spectroscopic methods showed binding constants in the range of 10^4^ to 10^5 ^M^−1^ for numerous drug-protein complexes, which are the same as the binding constants of PdNP to HSA [55]. The electrostatic force, van der Waals forces, hydrogen bonds, and hydrophobic interactions are among the non-covalent interactions that exist between ligands and proteins [56]. We used the van’t Hoff Eq. (Eq. (4)) to identify the thermodynamic parameters and main type of interaction between synthesized PdNP and HSA. This included the entropy variation (Δ*S*^°^) and enthalpy variation (Δ*H*^°^) of the binding reaction, which gave us data on fluorescence quenching [55].

4 $$lnK=-\frac{\Delta H^\circ }{RT}+\frac{\Delta S^\circ }{R}$$

where *K* refers to the binding constants at the appropriate temperature (*T*) derived from Eq. (3), and *R* represents the gas constant [55]. The following equation can be used to determine the values of free energy change (Δ*G*^°^) at various temperatures (Eq. (5)) [56].

5 $$\varDelta {\rm{G}}^\circ =\varDelta {\rm{H}}^\circ -{\rm{T}}\varDelta {\rm{S}}^\circ =-{\rm{RT}}\,\mathrm{ln}\,{\rm{K}}$$

The following are proposed values for the thermodynamic factors in the various protein–ligand interactions: Hydrophobic forces (a) Δ*H*^°^ > 0 and Δ*S*^°^ > 0, Vander Waals forces, hydrogen bonds (b) Δ*H*^°^ < 0 and Δ*S*^°^ < 0, and electrostatic contact (c) Δ*H*^°^ < 0 and Δ*S*^°^ > 0 [57]. For the interaction between produced PdNP and albumin protein, we determined the following values: Δ*S*^°^ (+0.24 kJ/mol.K), Δ*G*^°^ (−21.13 and −17.65 kJ/mol.K at 25 °C and 37 °C, respectively), and Δ*H*^°^ (+51.45 kJ/mol.K). At both temperatures, the Δ*G*^°^ values for the interaction between HSA and PdNP have a negative sign, indicating that the interaction is occurring spontaneously [58]. As another piece of evidence indicating hydrophobic interactions, positive values of the Δ*H*^°^ and Δ*S*^°^ for the binding of nanoparticles to HSA are typically accepted. However, it is possible that hydrophobic interactions are to blame for the presence of interactions between PdNP and HSA. It has been shown that palladium-based medicines or compounds attach to HSA through hydrophobic interactions. When [Pd(But-dtc)(phen)]NO_3_ (where But-dtc = butyldithiocarbamate and phen = 1,10-phenanthroline) interacted with HSA, Saeidifar et al. discovered that the palladium complex modified the secondary structure of HSA [59]. Moreover, research into the binding and structural modification of HSA by other metal-based drugs, such as platin complex (cis [Pt (NH_2_-Isopentylamine)2(Isopentylglycine)]NO_3_ [60] and silver nanoparticles (Ag-PVT) [61], reveals that these drugs can bind to and alter the structure of the HSA protein.

### Molecular dynamics (MD) simulations

**Fig. 6:**
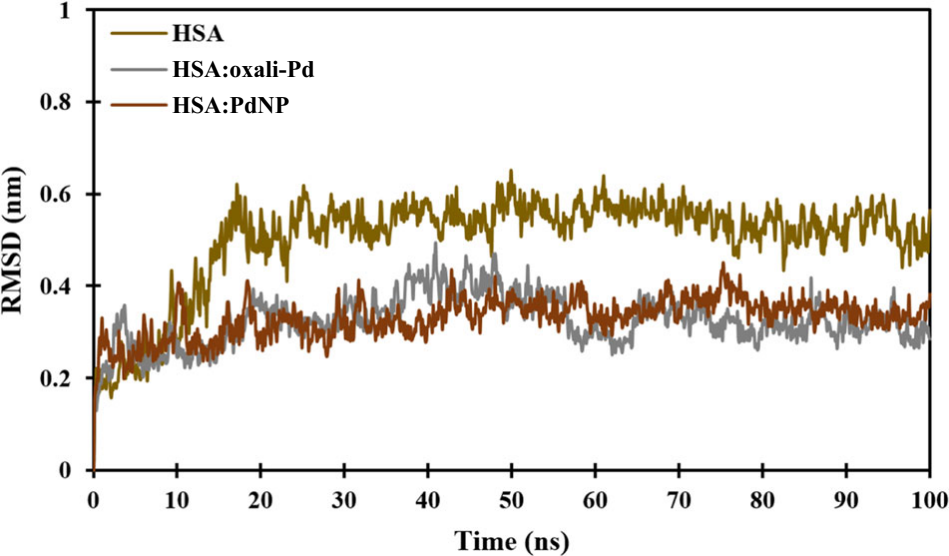
Atom-positional RMSD of the protein backbone in HSA, HSA:oxali-Pd, and HSA:PdNP

When evaluating the microstructure of proteins in real time, MD simulations are commonly utilized as a supplemental tool. As a bonus, we can gain several physicochemical parameters from these methods that would otherwise be time-consuming and expensive to obtain by experimental methods. To assess the dynamical behavior of unloaded HSA, HSA:oxali-Pd, and HSA:PdNP structures, we performed all-atom MD simulations using the strategy outlined in the “Materials and Method” section. Root-mean-square deviation (RMSD), solvent accessible surface area (SASA), the radius of gyration (*R*_g_), intermolecular hydrogen bonds, and interaction energies between proteins and ligands are some of the analytical tools being used to report the results. Throughout MD simulations, the mean distance between the backbone atoms of stacked proteins is measured by the root-mean-squared deviation (RMSD) of atomic locations. As a result, RMSD analysis might be used to investigate the simulated system’s equilibrium and stability. In addition, we reveal the RMSD of HSA backbone atom locations computed against the first structure of HSA, HSA:oxali-Pd, and HSA:PdNP (Fig. 6). Protein backbone RMSD levels decrease with time to roughly 0.53 nm for HSA, 0.31 nm for HSA:oxali-Pd, and 0.34 nm for HSA:PdNP, showing that the structures have reached equilibrium. A representative sample shows the systems’ structural and dynamical attributes that may be obtained after running the simulation for a sufficient time (Fig. 6).

**Fig. 7:**
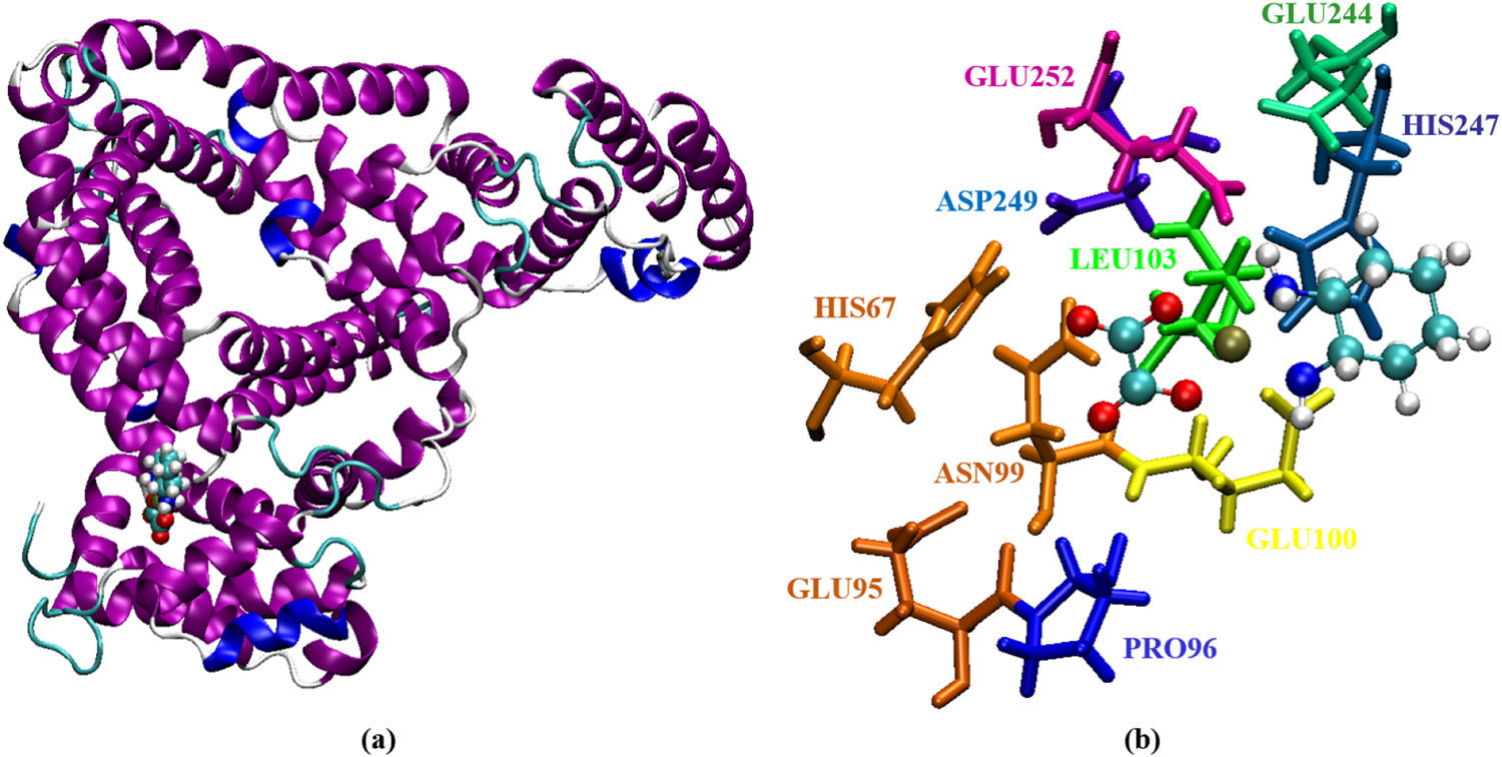
Representation of (**a**) the equilibrium conformation of the HSA:oxali-Pd complex and (**b**) amino acids that interact with the oxali-Pd molecule

**Fig. 8:**
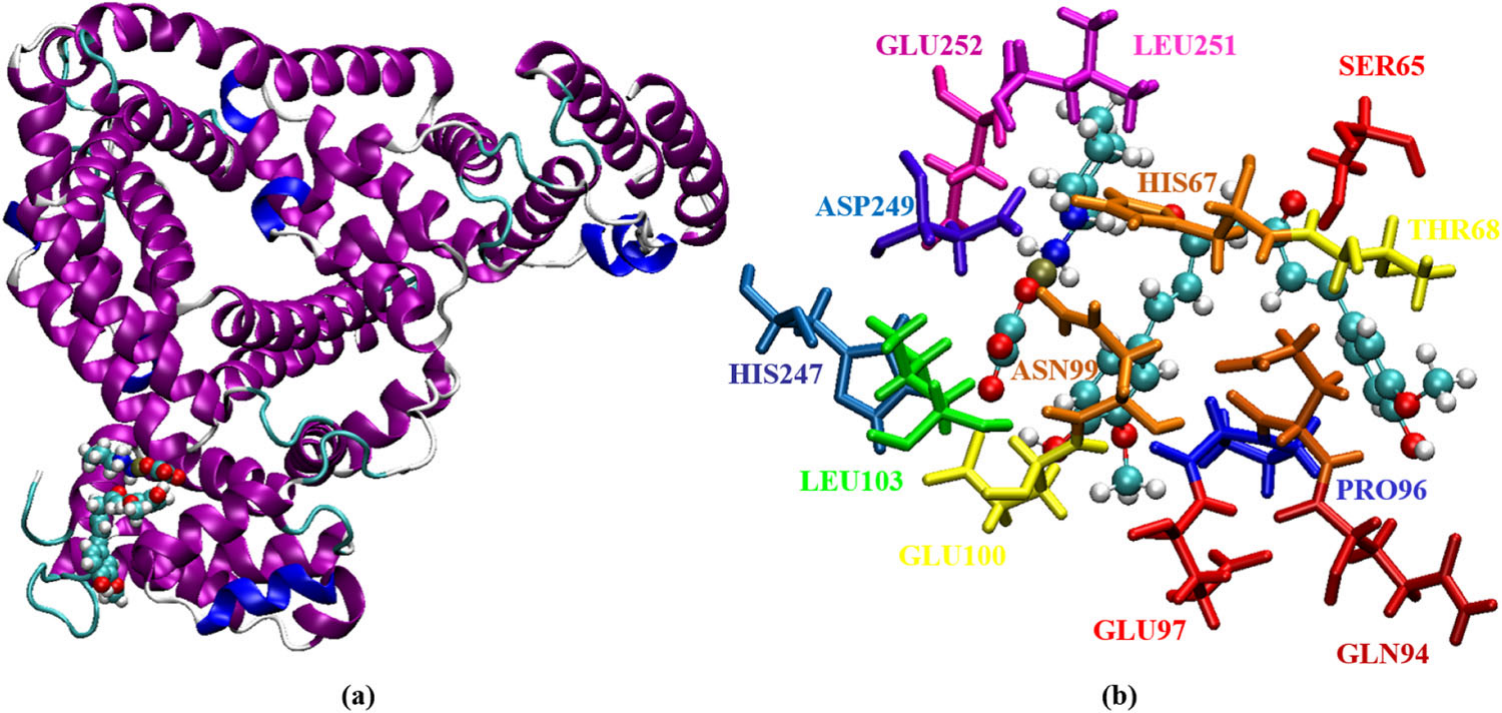
Representation of (**a**) the equilibrium conformation of the HSA:PdNP complex and (**b**) amino acids that interact with the oxali-Pd and CUR molecules

**Fig. 9:**
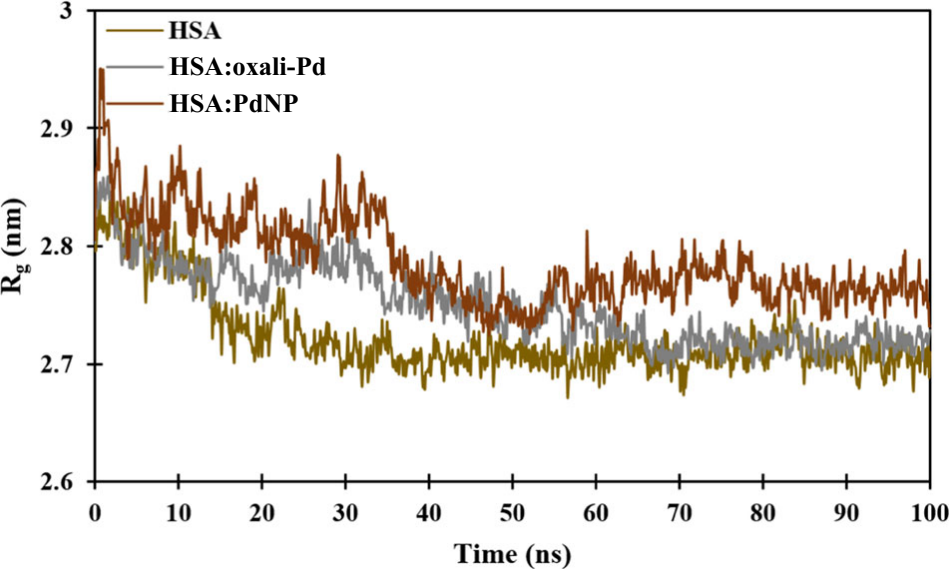
The time-dependent change in the HSA protein’s radius of gyration (*R*_g_) while unloaded and loaded

Here, we use a snapshot of the equilibrated conformation of HSA:oxali-Pd (Fig. 7a, b) and HSA:PdNP (Fig. 8a, b) to determine the binding sites preferred by each ligand. The residues HIS67, GLU95, PRO96, ASN99, GLU100, LEU103, GLU244, HIS247, ASP249, and GLU252 are all located within 5 Å of oxali-Pd (Fig. 7b). Interestingly, the preferential binding site of the drug molecule is unaffected by the addition of CUR to oxali-Pd and the formation of PdNP, so residues SER65, HIS67, THR68, GLN94, GLU95, PRO96, GLU97, ASN99, GLU100, LEU103, HIS247, ASP249, LEU251, and GLU252 all stabilize the HSA:PdNP complex (Fig. 8b). The following equation provides the radius of gyration (R_g_) for a molecular structure:

6 $${R}_{g}^{2}=\frac{1}{M}\mathop{\sum}\limits_{k=1}^{N}[{m}_{k}{({r}_{k}-{r}_{mean})}^{2}]$$

where *N* is the number of atoms, *M* is the total mass, *m*_*k*_ is the mass of the kth atom, *r*_*mean*_ is the average distance between the center of the molecule and the kth atom, and (*r*_*k*_ − *r*_*mean*_) is the distance between the kth and the center of the molecule. In this context, the *R*_g_ value describes the orientation of the protein’s atomic distribution. The distance *R*_g_ measures the radial distance from the center of rotation to the highest point of energy transfer. Thus, *R*_g_ gives a precise measure of protein compactness, as a firmly folded protein maintains a relatively constant *R*_g_ value throughout the MD trajectory, while an unfolded protein’s *R*_g_ value fluctuates. *R*_g_ of HSA was measured before and after interacting with oxali-Pd and PdNP (Fig. 9). Based on the final 10 ns of MD simulations, the average *R*_g_ values for HSA, HSA:oxali-Pd, and HSA:PdNP are 2.70 ± 0.01, 2.72 ± 0.01, and 2.79 ± 0.01 nm, respectively.

**Fig. 10:**
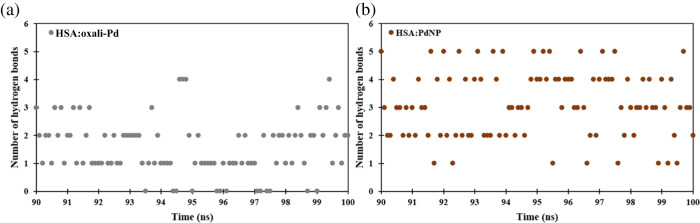
The number of intermolecular hydrogen bonds formed between (**a**) oxali-Pd and topo IIα protein during the MD simulations (**b**)

HSA must undergo structural changes to form a stable complex with PdNP, while oxali-Pd seems to bind into the -helix of HSA via the local alterations in protein cavities without affecting its shape or compactness. Since hydrogen bonds are one of the driving forces behind the stabilization of macromolecules like proteins, DNA, and RNA, studying intermolecular hydrogen bonds between proteins and ligands has long been of tremendous interest in complex stability research. To conduct hydrogen bond analysis, the bond length and angle cutoff were adjusted to 3.5 Å and 120^°^, respectively. Hydrogen bonds produced between HSA and ligands in HSA:oxali-Pd and HSA:PdNP complexes over the last 10 ns of MD trajectory are displayed (Fig. 10). Hydrogen bonds are created between HSA and both oxali-Pd and PdNP throughout the simulation. However, the average number of hydrogen bonds produced between HSA and PdNP (~3.2) is significantly higher than those formed between protein and oxali-Pd (about 1.5).

**Fig. 11:**
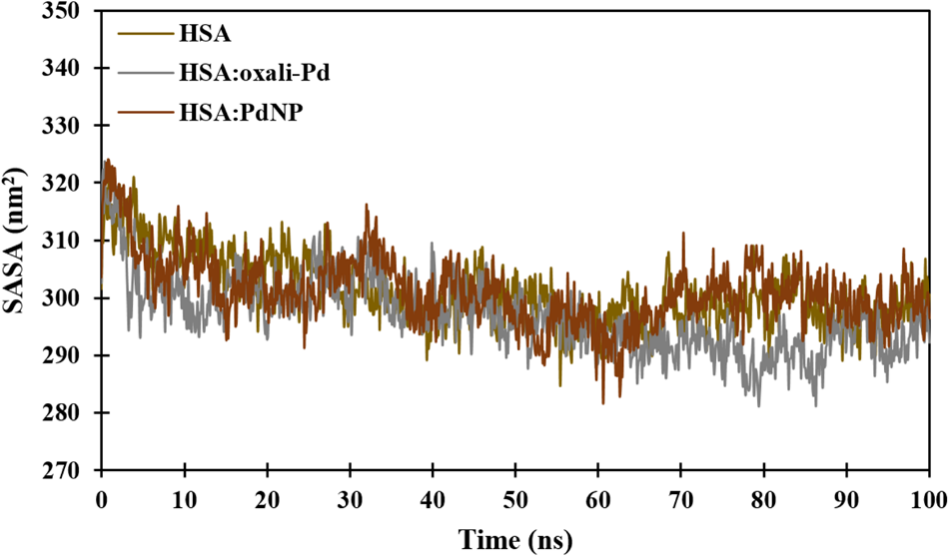
Solvent accessible surface area (SASA) as a function of simulation time for unloaded and loaded proteins

Protein stability and folding are thought to be largely determined by the protein’s solvent-accessible surface area (SASA). The van der Waals contact surface of a molecule is used to define this value, which is calculated by comparing the center of a hypothetical solvent sphere to the protein’s van der Waals contact surface (10.2174/1389203715666140327114232). It was necessary to determine how much of the HSA protein could be accessed by solvents, so a SASA analysis was performed. SASA values of roughly 298, 294, and 299 nm^2^ over the last 10 ns of MD simulations for HSA, HSA:oxali-Pd, and HSA:PdNP show that neither oxali-Pd nor CUR encapsulation causes a significant structural deformation of the protein (Fig. 11).

According to our calculations, the Coulombic and Lennard-Jones (LJ) energies for HSA: oxali-Pd are 65.8 ± 9.3 and −71.7 ± 11.4 kJ mol^−1^, respectively. In the case of the HSA:PdNP complex, these values rise to 194.1 ± 18.4 and 215.8 ± 21.7 kJ mol^−1^, respectively. These figures for the interaction energies shed light on the significance of electrostatic and dispersion stabilization in the HSA:oxali-Pd and HSA:PdNP complexes, respectively.

## Conclusions

Nanotechnology is an emerging field that holds promise as a novel approach to treating numerous diseases, including cancer. In laboratory studies, metal nanoparticles, specifically palladium, have shown cancer cell cytotoxicity. A nano-oxali-palladium coated with turmeric extract was successfully produced in this study, with a diameter of less than 100 nm and a spherical, homogenous shape. Using fluorescence quenching data, protein binding results demonstrated that PdNPs have a potent ability to quench and react with the HSA protein. The molecular dynamics simulations show that oxali-Pd attaches to the -helix of HSA via the local changes in protein hollow spaces without altering the protein’s shape or compactness. In contrast, PdNP requires HSA to undergo structural modifications to create a stable complex. Oxali-Pd and PDNP share the same preferential binding location. In the case of HSA:oxali-Pd, the computed coulombic and Lennard-Jones (LJ) interaction energies are 65.8 ± 9.3 and −71.7 ± 11.4 kJ.mol^−1^, respectively. In the case of the HSA:PdNP complex, these values rise to 194.1 ± 18.4 and 215.8 ± 21.7 kJ.mol^−1^. As a result, the complex is held together by both coulombic and LJ non-bonded forces. The produced nanoparticles exhibit higher cytotoxicity than free oxali-palladium and free extract and can induce apoptosis in the HCT116 colorectal cancer cell line. This study’s findings could lead to the conclusion that, in the future, palladium nanoparticles made using green chemical techniques would replace conventional treatment approaches, ushering in a new paradigm in oncotherapy.

## Supplementary information

Supplementary information

## Data Availability

The data presented in this study are available on request from the corresponding author.
