# Supplementary material for: Oxali-palladium nanoparticle synthesis, characterization, protein binding, and apoptosis induction in colorectal cancer cells
Source: J Mater Sci Mater Med. 2024 Jan 11;35(1):4. doi: 10.1007/s10856-023-06766-8 (PMC10784377; doi:10.1007/s10856-023-06766-8)
Supplement: Supplementary file 1 — Supplementary information [file 10856_2023_6766_MOESM1_ESM.docx]

**Figure S1.** Changes in UV–visible spectroscopy spectra of (a) free oxali-palladium (before reduction reaction, yellow colors solution). (b) Nano-oxali-palladium (after reduction reaction, brown color solution) after 24 hours at 50 ^°^C and 200 rpm in a shaking incubator.

**Table S1**. Differences and similarities in functional groups in free oxali-palladium and nano-oxali-palladium obtained from FTIR studies.

**Table 1**. Cc_50_ values for oxpd, oxpdNps, oxpt, and turmeric extract after 24 and 48 hours of incubation.

| **Cc_50_**  **After 48 h** | **Cc_50_**  **After 24 h** | **Compounds** |
| --- | --- | --- |
| 433 (μM) | 600 (μM) | Oxpd |
| 57 (μM) | 78 (μM) | Oxpd NPs |
| – | 1100 (μM) | Oxpt |
| 32 (mg/mL) | 45 (mg/mL) | Turmeric extract |

# Supplemental information

## Figure S1
